# Why does apical periodontitis persist? A shotgun metagenomic survey of the microbial community in primary and persistent periapical granulomas

**DOI:** 10.1080/20002297.2026.2741866

**Published:** 2026-10-28

**Authors:** Yuqing Shen, Yiling Yang, Jian Sun, Zhihui Han, Yang Sun, Ruixue Li, Yu Zhang, Youcheng Yu

**Affiliations:** a Department of Stomatology, Zhongshan Hospital, Fudan University, Shanghai, China; b Department of Stomatology, Xuhui Central Hospital, Fudan University, Shanghai, China; c Department of Preventive Dentistry, College of Stomatology, Shanghai Ninth People’s Hospital, Shanghai Jiao Tong University School of Medicine, Shanghai, China

**Keywords:** Persistent apical periodontitis, primary apical periodontitis, metagenomics, metagenomic sequencing, microbiota, periapical periodontitis, periapical granuloma

## Abstract

**Objective:**

This cross‑sectional observational study used shotgun metagenomic sequencing to compare the microbiomes of periapical granulomas from primary and persistent apical periodontitis to identify microbial taxa and functional pathways associated with persistent lesions.

**Methods:**

Seventeen patients with untreated primary apical periodontitis (UA), 19 with persistent apical periodontitis (PA), and 19 healthy gingival reference samples (HC) were enrolled. Periapical granuloma and healthy gingival samples were collected during tooth extraction for whole-metagenome shotgun sequencing. Alpha diversity was assessed by the Shannon and Chao1 indices. Beta-diversity was evaluated using PERMANOVA based on Bray‑Curtis distances. Differential abundance analysis of species and predicted functional pathways between UA and PA was performed using the Mann‒Whitney U test with Benjamini‒Hochberg false discovery rate (FDR) correction. Clinical associations were examined using Spearman’s correlation. The HC group served as a descriptive reference.

**Results:**

Alpha diversity showed a significant difference in species richness (Chao1, *p* < 0.05) but not overall diversity (Shannon, *p* > 0.05). Beta diversity revealed a trend toward separation but did not reach statistical significance (PERMANOVA, *R^2^* = 0.05085, *p* = 0.051). Metagenomic sequencing revealed differences in species composition and functional potential between UA and PA. Taxonomic profiling identified 503 species in UA versus 255 in PA. At the species level, several taxa showed nominal differences between UA and PA (*p* < 0.05), but none remained significant after FDR correction (*q* > 0.05). Functionally, several KEGG pathways showed nominal differences between UA and PA (*p* < 0.05, *q* > 0.05), with the top three pathways all below 3% relative abundance. Swelling duration correlated negatively with several taxa and pathways (*p* < 0.05), while lesion diameter showed little association with microbial abundance.

**Conclusions:**

Persistent apical periodontitis granulomas showed lower species richness and abundance, but similar predicted functional capacity, compared with primary infections. Further longitudinal and multi‑omics studies are needed to investigate the associations.

## Introduction

Apical periodontitis (AP) is an inflammatory disease caused by microbial infection in the root canal system. It is estimated that AP affects up to 52% of the worldwide population [1]. Approximately 50% of periapical lesions are classified as periapical granulomas [2]. Root canal treatment (RCT) aims to eliminate infection by removing necrotic pulp tissue and disinfecting the root canal system. RCT significantly alters the root canal microbiome, but its impact on extraradicular biofilms may be limited [3]. However, residual bacteria may be associated with persistent infections and are thought to play a contributory role in the chronic inflammatory process [4], which may potentially lead to treatment failure and bone loss. Despite advances in endodontic treatment, it remains unclear whether specific bacterial species that survive RCT are associated with persistent inflammation and periapical bone destruction [5]. Understanding the composition and diversity of the apical canal microbiota is therefore essential for developing effective therapeutic strategies.

Over 460 bacterial taxa are associated with infected root canals, but the precise composition of the endodontic microbiota remains incompletely understood owing to its remarkable complexity [6]. Conventional culture-based approaches have long limited the detection of pathogenic microorganisms, as they are unable to cultivate approximately 50% of oral bacterial species [7]. Although various identification techniques have shown that approximately 70% of these bacteria are obligate anaerobes or microaerophiles, the full diversity of this community has yet to be fully resolved [8].

Previous amplicon sequencing studies have compared the bacterial communities of primary and persistent apical periodontitis, revealing differences in community composition at the genus level using 16S rRNA [5,9]. However, this approach has two major limitations: first, it typically offers limited resolution at the species level due to high sequence similarity within many bacterial genera; second, it provides no information on the functional potential of the microbial community, as it relies on phylogenetic markers rather than whole-genome content [10]. These constraints hinder our ability to identify specific pathogenic species and to understand their predicted metabolic contributions to disease persistence.

Shotgun metagenomic sequencing directly addresses these limitations by enabling species‑level taxonomic assignment and simultaneous characterization of predicted functional gene potential via KEGG pathway analysis [11]. This method now plays a pivotal role in exploring how microbial communities may be related to oral disease, providing a more comprehensive basis for identifying candidate biomarkers and functional features associated with disease persistence.

Therefore, the present cross‑sectional observational study employed shotgun metagenomic sequencing to compare the microbial taxonomic and predicted functional profiles of periapical granulomas from primary and persistent apical periodontitis. We aimed to identify microbial taxa and functional pathways associated with persistent lesions and to explore potential correlates of clinical parameters.

## Materials and methods

### Ethical approval and registration

The cross‑sectional observational study was approved by the Zhongshan Hospital Ethics Committee (Protocol No: B2025-023) and registered at the Chinese Clinical Trial Registry (ChiCTR2500107795). All participants provided written informed consent after receiving a detailed explanation of the surgical procedures, possible discomforts, and their right to withdraw at any time. This manuscript was written according to the STROBE guidelines.

### Study design and grouping

Sample size calculations were estimated with reference to previously published metagenomic studies in endodontic microbiology [12], with a specific focus on the primary UA versus PA comparison. The sample size was determined according to the simulation-based framework for microbiome PERMANOVA proposed by Kelly et al. [13] using the R package *micropower*, with the following parameters: expected effect size (*ω^2^*) = 0.05, statistical power = 0.8, and significance level (*α*) = 0.05. This effect size was chosen based on the moderate effect sizes reported in comparable microbiome studies and was considered sufficient to detect meaningful differences in community composition between the two disease groups. Under these settings, PERMANOVA achieves > 80% power with as few as 15 subjects per group, which was the estimated number required per group for the UA vs. PA comparison. To account for potential sample degradation or processing loss, an additional 20% was added to the estimated requirement for each group, resulting in target sample sizes of approximately 18 per group. The HC group was similar to the PA group in terms of age, gender, and periodontal status. The final sample sizes were 19 for HC, 17 for UA, and 19 for PA, all of which exceeded the minimum requirement of 15 per group. The inclusion criteria and exclusion criteria are listed in Table 1.

**Table 1.** Inclusion criteria and exclusion criteria of the study.

Inclusion criteria	Exclusion criteria
(a) Patients aged 18 to 70 years, with an indication for tooth extraction based on clinical examination. (b) Voluntary participation with signed informed consent and provision of personal medical history. (c) No systemic or topical oral antibiotic therapy within the preceding three months. (d) Presence of at least 20 functional teeth, absence of rampant caries, oral mucosal disease, severe periodontal disease, acute inflammatory conditions, and removable dentures or appliances.	(a) Serious systemic conditions, including cardiac arrhythmias, uncontrolled hypertension, diabetes mellitus, hepatitis (any aetiology), and renal failure. (b) Pregnant or lactating women, or those planning pregnancy during the study. (c) Prior facial radiotherapy, or use of bisphosphonates, denosumab, or other antiresorptive medications. (d) Active smokers. (e) Periodontal treatment or antibiotic therapy within three months prior to enrollment. (f) Mental illness or any condition impairing decision-making capacity or legal competence.

The present study was conducted from January 2025 to December 2025 in the Department of Stomatology, Zhongshan Hospital, Fudan University, Shanghai, China. Fifty-five individuals scheduled for tooth extraction who met the inclusion/exclusion criteria were recruited and classified into three groups according to the preoperative diagnosis.

1. Healthy gingival reference group (HC, *n* = 19): asymptomatic impacted third molars extracted for orthodontic reasons. Excess healthy gingival tissue was harvested during surgery.
2. Untreated primary apical periodontitis group (UA, *n* = 17): teeth with untreated primary apical periodontitis were diagnosed clinically and radiographically, and were considered non-restorable by endodontic intervention, primarily attributable to extensive periapical pathology or insufficient remaining coronal structure.
3. Persistent apical periodontitis group (PA, *n* = 19): teeth that had received technically acceptable RCT at least 12 months earlier but still presented with persistent periapical radiolucency and/or clinical symptoms (e.g. sinus tract, swelling, or tenderness to percussion). To ensure the inclusion of true persistent disease, the quality of previous treatment was independently assessed by two endodontic specialists using preoperative periapical radiographs and CBCT. The treatment was considered technically acceptable only if the filling was within 0–2 mm of the apex, showed uniform density without voids, had adequate taper, no missed canals, no perforation or fracture, and an adequate coronal restoration without leakage [14]. Alternative treatments (retreatment or periapical surgery) were discussed with the patient, along with the risks, benefits, expected success rates, time commitment, and financial costs of each option. These teeth were extracted after an endodontic specialist determined that the prognosis was unfavourable, and the decision to extract was made in consultation with the patient.

All specimens from both disease groups (UA and PA) were obtained exclusively from extracted teeth using identical protocols to ensure methodological consistency in tissue acquisition. This standardized approach minimized technical variability between groups that might otherwise result from procedural variation (such as periapical surgery).

Two experienced dentists performed all the clinical examinations following a standardized protocol. Demographic data, including gender, age, comprehensive medical, and dental histories, were recorded. A full-mouth periodontal examination was conducted at six sites per tooth, recording the gingival index (GI: 0–3), plaque index (PI: 0–3), probing depth (PD), and decay-missing-filling teeth (DMFT) index. In the UA and PA groups, the duration of the current swelling episode was recorded for each patient, and the maximum diameter of periapical radiolucency was measured on Cone-Beam CT images.

### Sample collection

The samples were obtained according to the following procedure. First, patients rinsed with 0.2% chlorhexidine gluconate mouthwash for 30 s, and the perioral skin was disinfected with 5% povidone-iodine. After applying local anaesthesia and tooth extraction, the periapical granuloma attached to the root apex was carefully curetted from the socket using sterile surgical curettes in PA and UA groups. In HC group, excess healthy gingival tissue from the surgical site was collected using sterile scalpels. Strict aseptic techniques were maintained throughout the procedure to prevent contamination of the periapical tissue. All the samples were immediately flash-frozen in liquid nitrogen (−196 °C) and subsequently stored in −80 °C freezers for long-term preservation to prevent nucleic acid degradation. All procedures were performed using sterile gloves and instruments in a dedicated clean field. Following sample collection, all specimens were assigned coded alphanumeric IDs, and the group allocation key was maintained by a single investigator not involved in the laboratory procedures. The periapical specimens from the UA and PA groups were submitted for histopathological examination, which confirmed the diagnosis of periapical granulomas.

#### DNA extraction, sequencing, and metagenomic analysis

Technicians performing sample processing, DNA extraction, and sequencing were blinded to group allocation. Total genomic DNA was extracted from tissue samples using the FastPure Stool DNA Isolation Kit (Magnetic Bead; MJYH, Shanghai, China). The DNA concentration and purity were assessed by Synergy HTX and NanoDrop 2000, respectively, and the quality was verified on 1% agarose gel. DNA was fragmented to ~350 bp using Covaris M220 (Gene Company Limited, China) for paired-end library construction with NEXTFLEX Rapid DNA-Seq (Bioo Scientific, Austin, TX, USA). Sequencing was performed on Illumina NovaSeq X Plus (Illumina Inc., San Diego, CA, USA) using the NovaSeq X Series 25B Reagent Kit. The raw sequence data have been deposited in the Genome Sequence Archive (Genomics, Proteomics & Bioinformatics 2025) in the National Genomics Data Center (Nucleic Acids Res 2025), China National Center for Bioinformation/Beijing Institute of Genomics, Chinese Academy of Sciences (GSA-Human: HRA019020), which are publicly accessible at https://ngdc.cncb.ac.cn/gsa-human [15,16].

Negative extraction controls (no-template control) were included during DNA extraction and library preparation, and PCR amplification showed no detectable bands. Shotgun sequencing of an independent blank control sample yielded 0 kb, confirming no significant exogenous contamination.

Data analysis was performed on the Majorbio Cloud Platform (www.majorbio.com) [17], which performs analyses in a fully automated manner with default parameters, and analysts were blinded to group identities until the final taxonomic and functional tables were generated. The raw reads were trimmed and filtered (length < 50 bp or quality score < 20) using fastp (v0.20.0) [18]. Reads were aligned to the human genome by BWA (v0.7.17) [19], and any hit associated with the reads and their mated reads were removed. The quality-filtered reads were assembled using MEGAHIT (v1.2.9) [20], and contigs ≥ 300 bp in length were retained. Contigs with a length ≥ 300 bp were selected as the final assembly result. Open reading frames (ORFs) ≥ 100 bp were predicted using Prodigal (v2.6.3). A non-redundant gene catalogue was constructed using CD-HIT (v4.6.1) with 90% identity and coverage thresholds [21]. Gene abundance was estimated by SOAPaligner (v2.21) with 95% identity [22]. Taxonomic and functional annotations of non-redundant genes were generated by DIAMOND (v2.0.13) alignment against the NCBI NR database (e-value < 1e-5) [23]. The functional categories included the KEGG pathway.

### Quantitative PCR validation of selected taxa

Quantitative PCR (qPCR) was performed to validate the metagenomic findings for selected taxa. Sample DNA was extracted using the TIANGEN Bacteria DNA Kit (TIANGEN BIOTECH Bejing Co., Ltd). We used species-specific primers targeting *S. mutans (gtfB)* and *C. acnes (Pac)* with a QuantStudio™ 5 System. The universal 16S rRNA gene *(univ)* was used as an internal control. PCR amplifications were carried out in a total volume of 20  μL containing 10  μL of 2 × SYBR Green master mix, 0.5  μM of each primer, and 2  μL of template DNA (50 ng/μL). The amplification protocol consisted of initial denaturation at 95 °C for 15  min, followed by 40 cycles of 95 °C for 10  s, 60 °C for 20  s and 72 °C for 30 s. Melting curve analysis was performed to confirm specificity. All reactions were performed in triplicate. The primers used are listed in Table 2.

**Table 2.** Primers for qPCR.

	Forward sequence 5’-3’	Reverse Sequence 3’-5’
*univ*	TGGAGCATGTGGTTTAATTCG	TGCGGGACTTAACCCAACA
*gtfB*	GCCTACAGCTCAGAGATGCTATTCT	GCCATACACCACTCATGAATTGA
*Pac*	AGCTCGGTGGGGTTCTCTCATC	GCTTCCTCATACCACTGGTCATC

#### Statistical analysis

This study was designed primarily to investigate the differences between UA and PA groups, given that both groups represent the same tissue type. All formal hypothesis-driven analyses, including alpha/beta diversity comparisons, differential abundance testing and Spearman correlations with clinical parameters were restricted to these two groups. The HC group was included for descriptive reference and as a reference category in exploratory analyses of compositional profiles.

Baseline demographic characteristics were analyzed using SPSS (version 20.0, IBM, NY, USA), with statistical significance set at *α = 0.05*. Demographic data, including gender, age and clinical indices (GI, PI, PD and DMFT), along with disease duration and the maximum diameter of periapical radiolucency, were compared between the UA and PA groups. The normality of continuous variables was assessed using the Shapiro‒Wilk test, and the homogeneity of variances was evaluated using Levene’s test. For two‑group comparisons, Student’s t‑test was used when both normality and equal variance assumptions were met; otherwise, the Mann‒Whitney U test was applied. For categorical variables, the chi‑square test was used, with Fisher’s exact test applied when expected cell frequencies were less than 5. All tests were two‑sided, and a *p*‑value < 0.05 was considered statistically significant.

Alpha diversity was evaluated with a bias-corrected Chao1 richness estimator, and the Shannon diversity index was calculated through Mothur [24]. Beta diversity was visualized using principal coordinates analysis (PCoA) based on Bray-Curtis distances. Differences in community composition between groups were assessed using PERMANOVA (999 permutations). The homogeneity of multivariate dispersion was evaluated using the PERMDISP to determine whether significant PERMANOVA results could be attributed to differences in group dispersion rather than group centroids.

Characteristic sequences were classified across multiple taxonomic levels from phylum to species. Intergroup comparisons employed the Mann–Whitney U tests with a fixed *α* value of 0.05 between UA and PA. For differential abundance analyses involving multiple taxa and predicted functional pathways, all *p*‑values were adjusted for multiple comparisons using the Benjamini‒Hochberg false discovery rate (FDR) method, and an FDR‑adjusted *q* < 0.05 was considered statistically significant.

LEfSe (Linear Discriminant Analysis Effect Size) was performed as an exploratory discriminant analysis with a logarithmic LDA score threshold of 3.0. LEfSe results are interpreted as hypothesis‑generating. The HC group was included as a reference category for ranking purposes but was not incorporated into any formal pairwise hypothesis testing, which was restricted to the UA vs. PA comparison. These findings were not subject to formal multiple testing correction and should be interpreted as candidate biomarkers requiring further validation.

Functional annotation of the genome was conducted using level-3 pathways according to the Kyoto Encyclopedia of Genes and Genomes (KEGG) database for comprehensive metabolic and functional analysis [25]. Species and functional contribution analyses were performed to evaluate the discriminatory power of individual bacterial taxa and pathways between UA and PA.

Spearman's rank correlation was used to evaluate associations between clinical parameters and microbial abundance, as well as with KEGG pathway abundance (level 3). Heatmaps were generated using the *pheatmap* package in R (v3.3.1).

## Results

### General characteristics of participants

A total of 55 patients were included in this study and classified into 3 groups: healthy gingival reference group (HC, *n* = 19, 7 males and 12 females; mean age: 31.76 ± 7.45 y), untreated apical periodontitis group (UA, *n* = 17, 10 males and 7 females; mean age: 38.59 ± 12.00 y), and persistent apical periodontitis group (PA, *n* = 19, 9 males and 10 females; mean age: 36.11 ± 8.86 y). The baseline demographic and clinical characteristics of the study population are summarized in Table 3 for the three groups. Comparisons between UA and PA showed no significant differences for any of the variables examined. Age and clinical indices (GI, PI, PD, and DMFT) were analyzed using the Mann‑Whitney U test, while gender distribution was compared using the chi‑square test. Disease duration and the maximum diameter of periapical radiolucency were compared using Student’s t‑test. All *p* > 0.05 (Table 3).

**Table 3.** Demographic and clinical comparability of UA and PA groups.

	Group			UA vs PA	
	HC	UA	PA	*χ^2^/Z/t*	*p*
Gender (Male: Female)	7: 12	10: 7	9: 10	0.63	0.525
Age	31.76 ± 7.45	38.59 ± 12.00	36.11 ± 8.86	154	0.306
PD (mm)	2.99 ± 0.63	3.04 ± 0.56	3.03 ± 0.54	161	0.500
DMFT	1.49 ± 0.22	2.65 ± 1.50	3.00 ± 1.29	134.5	0.195
PLI	1.76 ± 0.93	1.56 ± 0.95	1.67 ± 0.84	150	0.364
GI	1.04 ± 0.61	1.13 ± 0.60	1.67 ± 0.65	151	0.376
Duration	/	2.44 ± 1.25	3.16 ± 1.07	−1.86	0.072
Diameter (mm)	/	5.79 ± 2.90	5.39 ± 2.41	0.45	0.654

### Characterization of microbiota

#### Alpha and beta diversity at the species level

Metagenomic sequencing was successfully conducted on all the samples from the three groups. Alpha diversity analysis showed that species richness (Chao1 index) differed significantly between UA and PA (Figure 1a, *p* = 0.04762), whereas overall diversity (Shannon index) was not statistically significant (Figure 1b, *p* = 0.5058). PCoA revealed a trend toward separation between UA and PA groups (Figure 1c). PERMANOVA confirmed that this separation did not reach statistical significance (*R^2^* = 0.05085, *p* = 0.051). The PERMDISP test indicated that multivariate dispersion was comparable between groups (*p* = 0.10).

**Figure 1.**
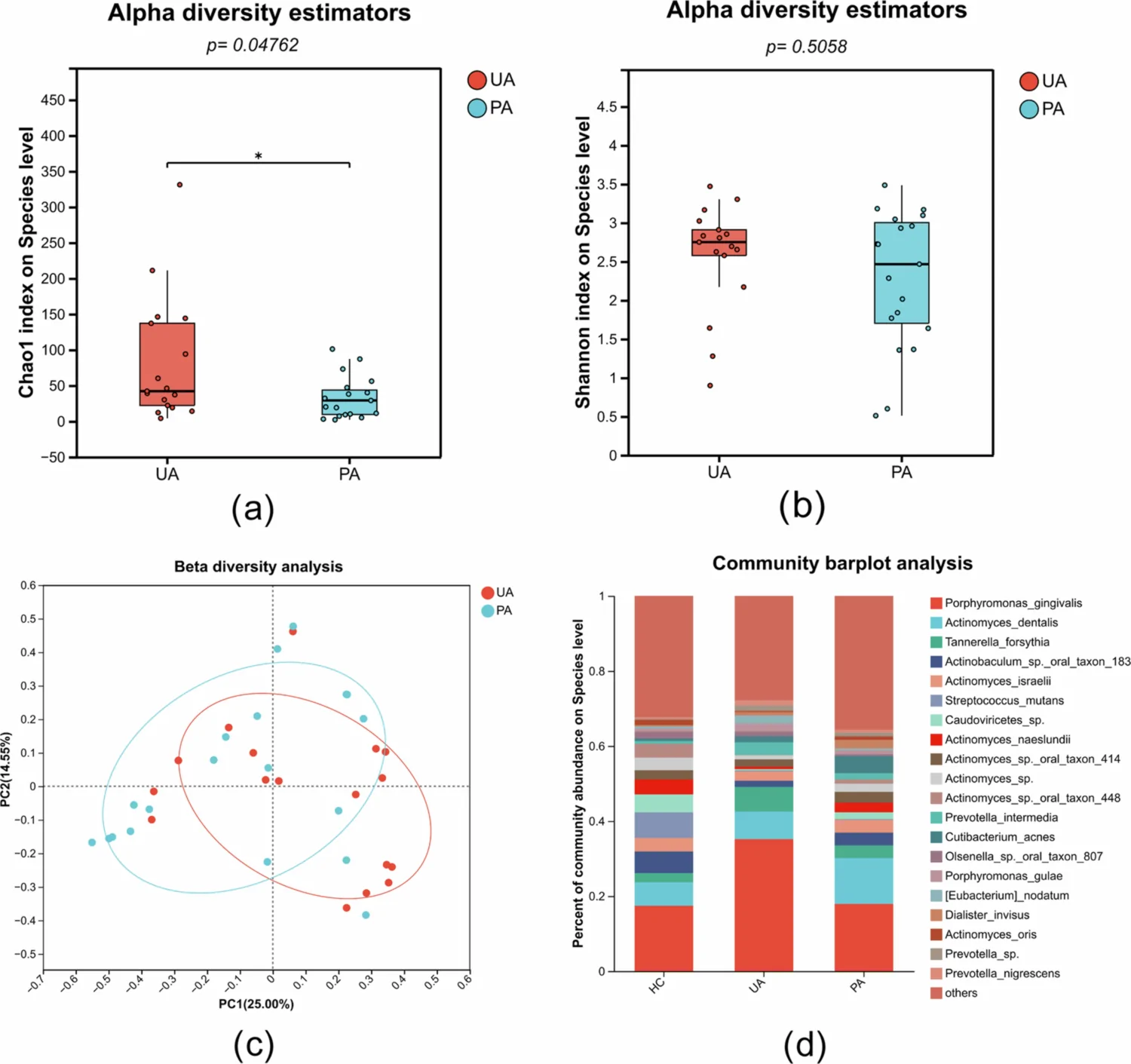
Alpha and beta diversity at the species level: (a) Chao1 index, (b) Shannon index and (c) PCoA plot based on Bray–Curtis distances showing the microbial community composition in UA and PA groups. The ellipses indicate 95% confidence intervals. (d) Bar plot showing the overall taxonomic composition at the species level across the HC, UA and PA groups.

#### Species composition

After data redundancy, 38 phyla, 67 classes, 104 orders, 173 families, 332 genera and 759 species were obtained from the metagenome data set. The overall taxonomic composition at the species level across the HC, UA and PA groups is depicted in Figure 1d. The HC group contains 34 phyla, 61 classes, 94 orders, 152 families, 251 genera and 562 species, while the UA group contains 20 phyla, 37 classes, 55 orders, 95 families, 190 genera and 503 species, and the PA group contains 15 phyla, 26 classes, 38 orders, 61 families, 102 genera and 255 species.

At the species level, *Porphyromonas gingivalis* was the most abundant species across all three groups. The subsequent species were *Streptococcus mutans* and *Actinomyces dentalis* in the HC group; *Actinomyces dentalis* and *Tannerella forsythia* in the UA group; and *Actinomyces dentalis* and *Cutibacterium acnes* in the PA group. An overview of the taxonomic data is presented in Supplementary Figure S1 (Phylum-Species).

A Venn diagram was plotted to visualize shared and unique taxa across HC, UA and PA groups (Figure 2). A total of 197 species were shared among all three groups. The HC group exhibited the highest number of exclusive taxa (*n = 225*), followed by UA (*n = 158*) and PA (*n = 12*).

**Figure 2.**
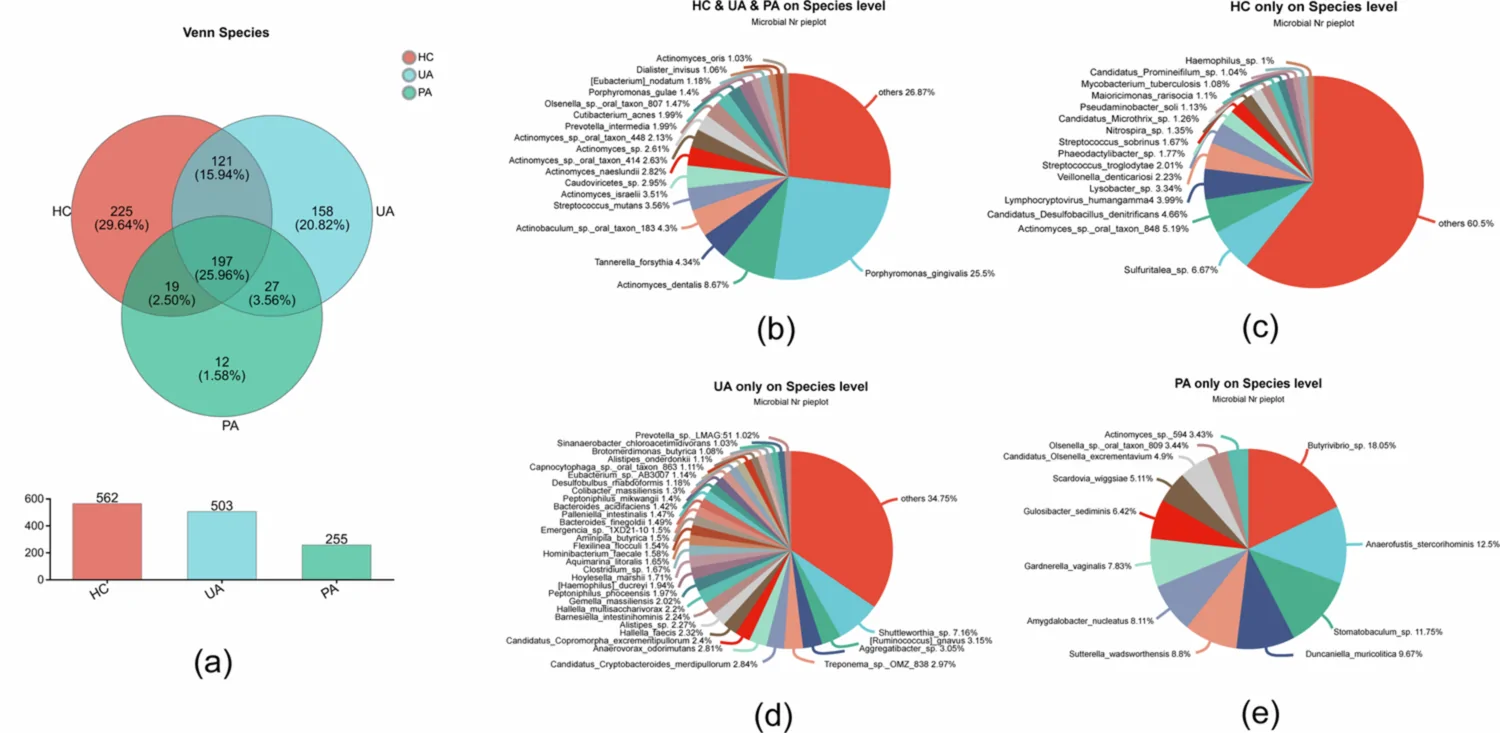
Venn diagram and pie chart analysis showing unique and shared bacterial species across HC, UA and PA groups. (a) A Venn diagram illustrating the number of unique and shared species among the three groups. Pie charts represent the relative abundance or proportion of taxonomic categories within each subset: (b) all three groups; (c) HC group; (d) UA group; (e) PA group.

#### Taxonomic differences

The top 20 most abundant species in the UA and PA groups are presented in Figure 3a. Mann-Whitney U test identified 4 significant differences in species abundance between UA and PA groups: *Prevotella intermedia* (*p* = 0.017), *Filifactor alocis* (*p* = 0.022), *Prevotella sp.* (*p* = 0.023) and *[Eubacterium] nodatum* (*p* = 0.021) showed higher abundance in the UA group. Notably, *Cutibacterium acnes* showed the higher abundance in the PA group, but the difference was not statistically significant compared to UA (*p* = 0.081). The raw *p*‑values and 95% *CI*s are shown in Figure 3a, while FDR-adjusted *q*‑values and effect size *r* are provided in Supplementary Table S1. However, after Benjamini‒Hochberg FDR correction, none remained significant (*q* > 0.05).

**Figure 3.**
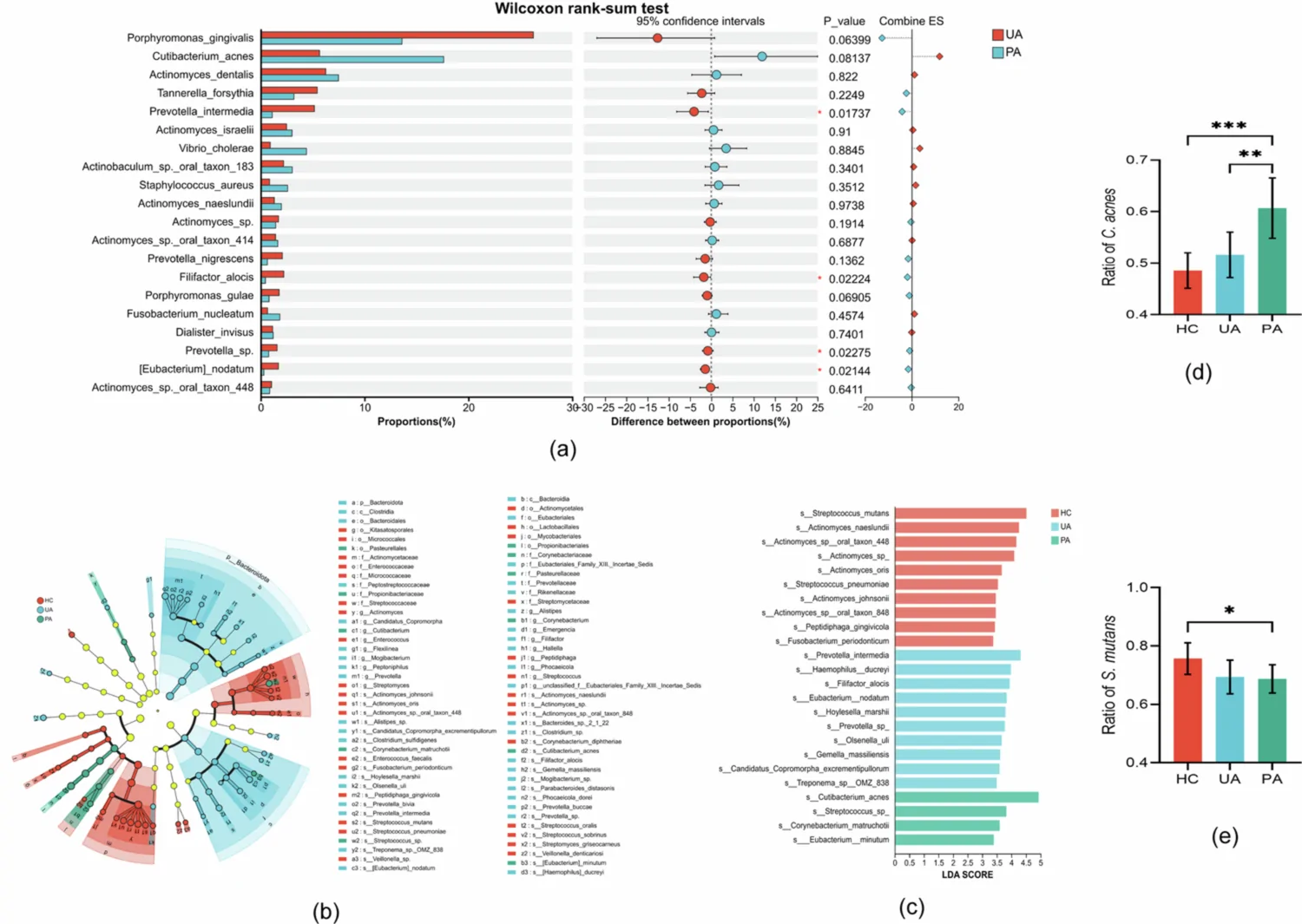
Differential abundance analysis. (a) Comparison of the top 20 abundant taxa in UA and PA using the Mann–Whitney U test (raw *p* < 0.05, with 95% *CI*, all *q* > 0.05). Exploratory LEfSe analysis (b) Taxonomic cladogram showing the hierarchical distribution of discriminative taxa with LDA scores > 3.0. (c) LDA score plot of group‑enriched taxa. Quantitative PCR verification of (d) *Cutibacterium acnes* and (e) *Streptococcus mutans.*

Exploratory LEfSe (linear discriminant analysis effect size) analysis was performed to rank taxa by their discriminative power across the three groups, with HC included as a reference category (Figure 3b and c). A total of 83 taxa showed nominal differences (*p* < 0.05), of which 26 exhibited strong discriminative potential (LDA > 3.0). Descriptively, HC was characterized by *Streptococcus mutans* and *Actinomyces spp.*; UA by *Prevotella intermedia* and *Filifactor alocis*; and PA by *Cutibacterium acnes* and *Corynebacterium matruchotii*. These findings are hypothesis‑generating and require validation in independent cohorts.

To further examine the differential abundance patterns, we performed qPCR for two relatively high-abundance and significantly different taxa: *Streptococcus mutans* and *Cutibacterium acnes*. The qPCR results were consistent with the sequencing data (Figure 3d and e). These qPCR data provide ancillary supportive evidence for the taxonomic profiles derived from shotgun metagenomic sequencing, rather than a comprehensive validation of all metagenomic findings.

### Gene function prediction

Functional annotation against the KEGG database revealed that the most abundant pathways across all three groups were ‘Metabolic pathways’ (ko01100), ‘Biosynthesis of secondary metabolites’ (ko01110) and ‘Microbial metabolism in diverse environments’ (ko01120), suggesting that core metabolic functions constitute the primary functional repertoire of the microbiome (Figure 4a).

**Figure 4.**
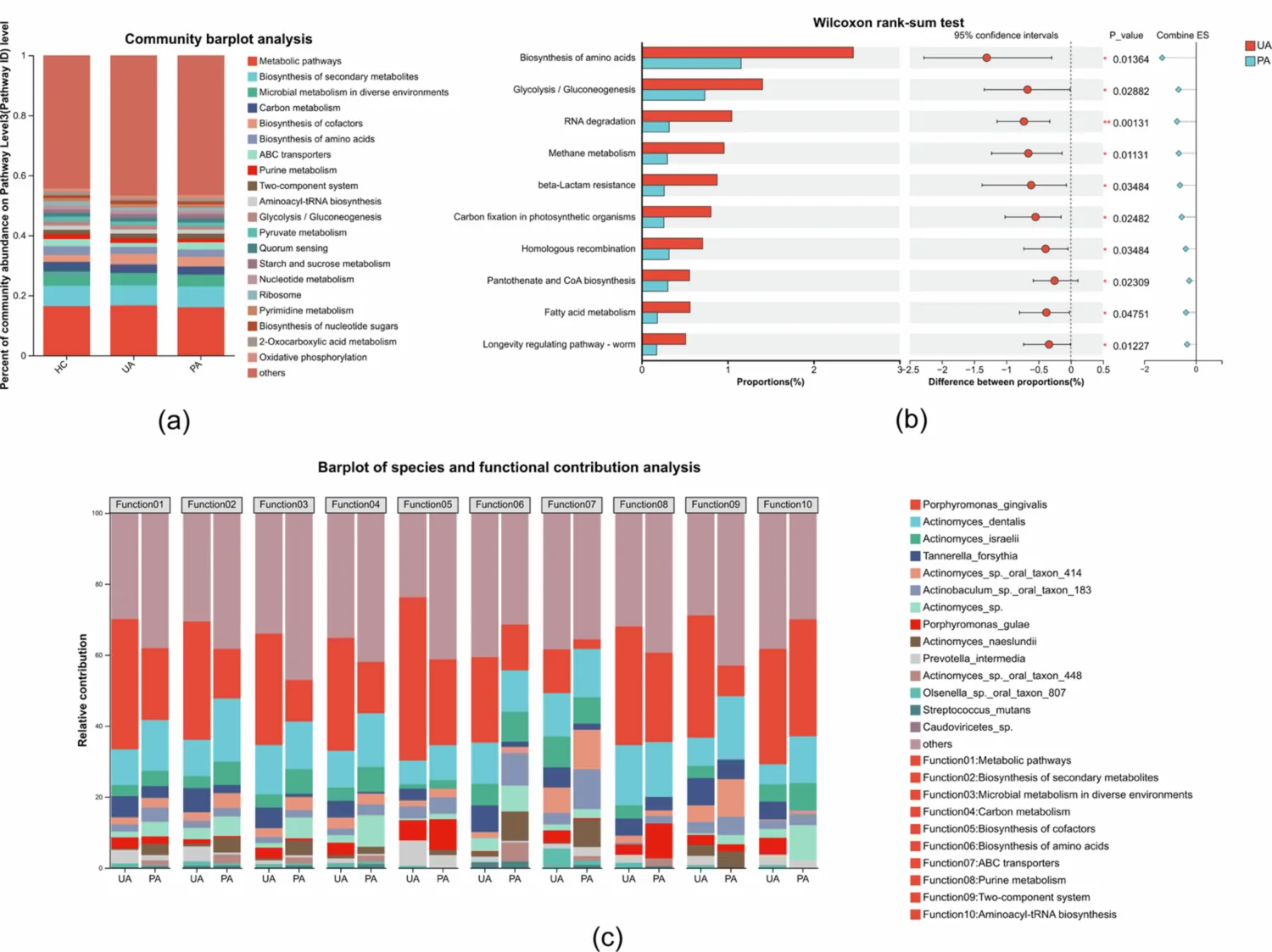
KEGG functional pathway analysis at Level 3 across HC, UA and PA groups. (a) Bar chart showing the top 50 most abundant functional categories, with the relative proportion of each function displayed. The low-abundance functions were grouped as ‘Others’. (b) Top 10 differentially abundant KEGG pathways identified by Mann–Whitney U test in UA and PA groups (raw *p* < 0.05, with 95% *CI*, all *q* > 0.05). (c) Species and function contribution analysis: contribution of species to specific KEGG pathways.

Mann–Whitney U test identified the differentially abundant KEGG pathways between UA and PA (Figure 4b). The three most abundant functional categories were ‘Biosynthesis of amino acids’ (ko01230), ‘Glycolysis/Gluconeogenesis’ (ko00010), and RNA degradation (ko03018), followed by ‘Methane metabolism’ (ko00680). All of these pathways were less abundant in the PA group than in the UA group, all with proportions less than 3%. However, after FDR correction for multiple comparisons, none remained significant at *q* < 0.05 (Supplementary Table S2).

To evaluate the contribution of individual bacterial species and functional pathways to the discrimination between UA and PA, species and functional contribution analyses were performed (Figure 4d). At the species level, the top five contributors in descending order were *Porphyromonas gingivalis, Actinomyces dentalis, Actinomyces israelii, Tannerella forsythia*, and *Actinomyces sp. oral taxon 414.* At the functional level, the top three contributing KEGG pathways were ‘Metabolic pathways’ (ko01100), ‘Biosynthesis of secondary metabolites’ (ko01110), and ‘Microbial metabolism in diverse environments’ (ko01120). These pathways showed substantially higher contribution scores than the others, suggesting that core metabolic functions and environmental adaptation were the primary functional signatures distinguishing between the two groups.

### Environmental factors associated with the bacterial community and functional pathways

Swelling duration demonstrated moderate negative correlations with *Porphyromonas gulae* (*ρ = −0.440, p = 0.0072*), *[Eubacterium] nodatum* (*ρ = −0.507, p = 0.0016*) and *Alloprevotella tannerae* (*ρ = −0.507, p = 0.0016*) (Figure 5a). Lesion diameter only showed a weak positive correlation with the relative abundance of *Parvimonas micra* (*ρ = −0.376, p = 0.0239*).

**Figure 5.**
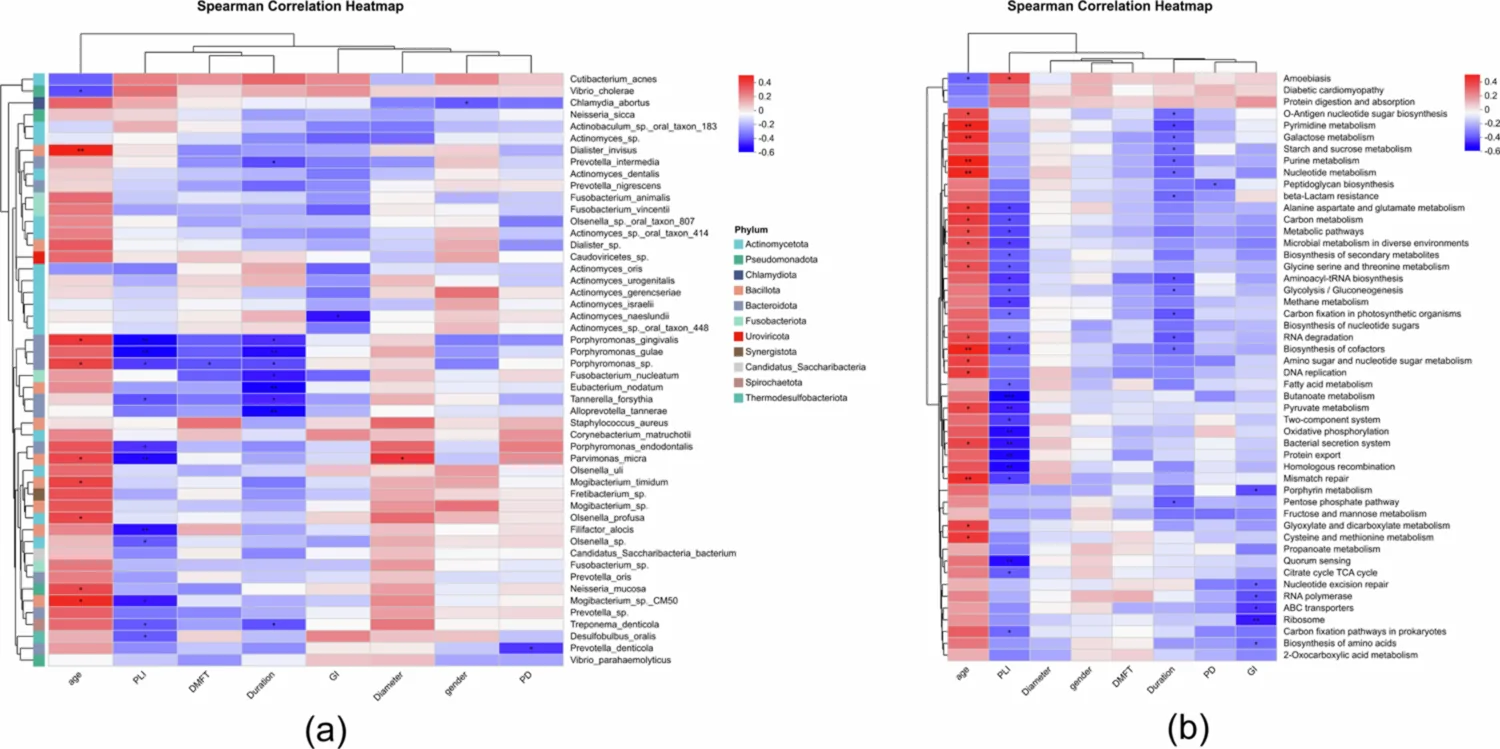
Spearman correlation analysis between clinical parameters and (a) microbial community structure, and (b) KEGG level 3 functional pathways. The clinical variables included age, gender, DMFT, GI, PI, PD, swelling duration and periapical lesion diameter. *p < 0.05*.

At the functional level, swelling duration was weakly but significantly negatively correlated with the abundance of multiple KEGG pathways (all *p* < 0.05, Figure 5b). These pathways encompassed core carbon metabolism (ko00010, ko00030, ko00052 and ko00720), nucleotide metabolism (ko00230, ko00240 and ko01232), biosynthesis pathways (ko00500, ko00540, ko00970 and ko01240), and information processing/other pathways (ko03018 and ko01501).

## Discussion

Persistent apical periodontitis has long been a difficult problem for clinical dentists. Current evidence suggests that persistent infection within and beyond the root canal system is one of the major contributors to disease persistence [4]. In this cross‑sectional shotgun metagenomic study, we compared the taxonomic and predicted functional profiles of periapical granulomas between primary and persistent apical periodontitis, with healthy gingival tissue included as a descriptive reference. Persistent lesions showed lower species richness and microbial abundance, but similar predicted functional capacity. Clinical parameters were also associated with microbial composition. These associations should be considered exploratory and warrant further investigation.

### Microbial composition

High-throughput sequencing methodologies, such as 16S rRNA gene amplicon sequencing, have markedly advanced our understanding of the microbial ecology of apical periodontitis. Several studies have indicated that microbial communities in both primary and persistent lesions, whether sampled from within the root canal or the surrounding periapical tissue, exhibit considerable diversity with marked interindividual variation [26–28].

In the present study, alpha diversity analysis showed that species richness differed significantly between UA and PA, while overall diversity remained similar. Beta diversity analysis revealed a trend toward separation between the two groups, although this difference did not reach statistical significance. This pattern may be consistent with the notion that root canal treatment could reduce microbial diversity, possibly through the elimination of taxa susceptible to antimicrobial procedures [29]. In our study, the PA group harboured considerably fewer observed species than did the UA group (255 vs. 503 species). This finding is consistent with previous findings that persistent apical periodontitis is a polymicrobial infection but contains many fewer species than the primary infection [30]. Whether this lower richness reflects treatment‑related elimination of taxa or other factors cannot be determined from the current cross‑sectional data. Longitudinal studies would be required to assess the ecological impact of root canal treatment on microbial communities.

Next-generation sequencing (NGS) is particularly effective for detecting low-abundance species-level taxa. Previous studies employing this technology have reported wide variation in species richness for primary apical canal infections: 37 (range, 13–80) [31]; 44 (range, 13–111) [32]; and 87 (46–184) [33]. Two NGS-based studies reported on the species richness in the apical canal of teeth with posttreatment: Siqueira observed a mean of 116 taxa per canal [34], while Ping [35] reported as many as 418 taxa, with a range of 235–654.

The higher species count observed in our study compared with previous reports may be attributed to the advancement of sequencing technology, which enables the detection of more species, particularly those with low abundance or those previously overlooked owing to technical limitations. Furthermore, the continuous expansion and curation of reference databases are critical for accurate taxonomic assignment [36].

### Microbial signatures of persistent disease

Previous studies have shown that primary periapical infections were dominated by *Fusobacterium*, *Lactobacillus*, *Prevotella*, *Actinomyces*, *Streptococcus*, *Parvimonas*, *Pseudoramibacter*, *Pyramidobacter* and *Porphyromonas* [31–33,37], while persistent endodontic infections include a high prevalence of *Enterococcus*, *Fusobacterium*, *Streptococcus*, *Actinomyces* and *Pseudoramibacter* [5,27,34,35,37]. The composition of the bacterial communities identified in the current study was broadly similar to those found in previous studies by 16S rRNA amplicon sequencing at the genus level, with *Porphyromonas* showing the highest proportion in UA and *Actinomyces* in PA.

By using shotgun metagenomic sequencing, we achieved species-level taxonomic assignment. The present study, therefore, benefits from a more detailed understanding of the microbial communities. At the species level, *Porphyromonas gingivalis* and *Actinomyces dentalis* were the two most abundant species in both groups, followed by *Tannerella forsythia* and *Prevotella intermedia* in the UA group, while *Cutibacterium acnes* and *Actinobaculum sp. oral taxon 183* in the PA group.

Comparative analysis revealed that *Prevotella intermedia* displayed the most significant intergroup abundance difference, with lower relative abundance in the PA group. *Prevotella intermedia* is a Gram-negative obligate anaerobe that has been consistently associated with symptomatic apical periodontitis and acute endodontic infections [9] and with potent proteolytic activity previously linked to symptomatic apical periodontitis [38]. The lower abundance in PA may suggest an association between *Prevotella intermedia* and post-treatment status, though its clinical relevance in failed RCT cases remains to be determined. Although the difference was not significant after FDR correction (*q* = 0.467), the observed trend of lower abundance in PA may warrant further investigation in larger cohorts.

*Cutibacterium acnes* (formerly *Propionibacterium acnes*) showed higher abundance in the PA group compared to the UA group, although the difference did not reach statistical significance (*p* = 0.081, *q* = 0.467). Interestingly, this trend was consistent with our qPCR findings. While *Cutibacterium acnes* is widely recognized as a common laboratory contaminant typically introduced from the skin, it is also plausible that its presence in the PA group may be associated with intraoperative introduction during RCT procedures, and may subsequently persist in the root canal system owing to its known tolerance to environmental stress and antimicrobial agents [39]. *Cutibacterium acnes* is believed to be involved in many infectious diseases of bones, joints, the eye and the brain, in addition to device-related infections [40].

An assessment of sodium hypochlorite and calcium hydroxide as antimicrobial agents in 24 necrotic root canals demonstrated that *Cutibacterium acnes* and *Streptococcus spp.* were still found after 7 days of treatment, whereas the same treatment was very effective at controlling other bacterial taxa [41]. We also acknowledge that separate environmental controls (e.g. air sampling in the operating field) were not included in the present study, which limits our ability to definitively distinguish contamination from true colonization. However, this remains speculative, and whether the presence of *Cutibacterium acnes* in treated cases correlates with clinical outcomes would require further investigation.

However, it should be noted that after FDR correction for multiple comparisons, none of the taxa remained significantly different between UA and PA, underscoring the need for validation in larger independent cohorts. Also, the taxa identified by LEfSe should be considered exploratory candidate markers rather than validated predictors, given the exploratory nature of the analysis and the relatively small sample size.

Additionally, among the top-20 taxa with no significant intergroup differences, *Actinomyces* displayed a relatively higher relative abundance, which may suggest a potential resistance to standard disinfection protocols. *Actinomyces* species have been implicated frequently as a cause of endodontic failure because of their ability to persist in periapical tissues [35,37]. Given the cross-sectional design of our study, we cannot distinguish between these possibilities. Longitudinal studies are warranted to clarify the role of Actinomyces in post-treatment outcomes.

### Predicted functional characteristics

KEGG pathway prediction indicated that the microbial functional repertoire was dominated by general metabolism-related pathways (ko01100, ko01110 and ko01120). Several pathways differed between the groups, notably ko01230, ko00010, ko03018 and ko00680; however, none remained significant after FDR correction (*q* > 0.05), and all remained below 3% relative abundance.

Interestingly, although the PA group showed less taxonomic diversity and abundance compared to the UA group, the overall functional gene composition remained relatively stable. A concept introduced in microbiome research is functional redundancy [42]. This theory states that functional profiles (i.e. proteolytic activity) could remain remarkably conserved despite the taxonomic diversity across individuals. It is also accepted that bacteria from the same species can present different pathogenic traits [43].

However, as these findings are based on genomic predictions rather than direct measurements of metabolic activity, they should be interpreted with caution and validated by metatranscriptomic or metabolomic approaches.

### Association between clinical parameters and microbial features

Previous studies have shown a positive correlation between the number of microbial taxa within the root canal and periapical lesion size. Canals associated with small lesions (<5 mm) harboured a mean of 11.7 taxa, those with lesions measuring 5–10 mm contained 16 taxa, and canals with lesions exceeding 10 mm yielded approximately 20 species [44]. The differences in species diversity between different lesion sizes help to explain the long-held concept that root canal treatment of teeth with large lesions has a lower success rate than treatment of teeth with small or no lesions [45]. More severe disease conditions can negatively impact the outcome and should present symptoms' resolution after initial debridement of the root canal space and prior to treatment completion. However, our study did not identify a significant correlation between lesion diameter and microbial diversity or functional composition. One possible explanation is that our sample consisted exclusively of periapical granulomas, which may have limited the range of lesion sizes available for analysis.

Swelling duration was negatively correlated with *Porphyromonas gulae, [Eubacterium] nodatum, Alloprevotella tannerae*, and multiple core metabolic pathways, suggesting lower predicted functional capacity in samples from patients with longer symptom duration. Given the modest strength of these correlations, these findings should be considered exploratory rather than confirmatory.

An exploratory random forest analysis was performed to assess potential predictors, but owing to the limited sample size and exploratory nature, the results are presented in the Supplementary Material (Supplementary Figure S1) and should be interpreted with caution.

### Limitations and clinical relevance of the study

Several limitations of this study should be acknowledged. First, the cross-sectional design precludes causal inferences. Our findings represent associations rather than causal relationships, and we cannot determine whether the observed microbial shifts precede or follow disease chronicity. Second, although consecutive recruitment was employed to minimize selection bias, the single-centre design and the inclusion of patients who underwent tooth extraction rather than conservative treatment may limit generalizability and introduce selection bias, as extraction cases may represent more severe or refractory disease. And third, while our specimens were histologically confirmed as granulomas, this also implies that our findings are specifically applicable to periapical granulomas and may not be generalizable to other periapical pathologies, such as cysts or abscesses. Since clinical and radiographic examinations cannot reliably distinguish granulomas from radicular cysts, scar tissue, or other periapical lesions, histopathological confirmation is essential for accurate classification of periapical tissue samples in studies of this nature.

The HC group was included as a descriptive reference. Healthy gingival tissue from impacted third molar extractions was selected as the reference because it is the most accessible, ethically obtainable healthy oral soft tissue, providing a practical reference for microbial comparison. However, the interpretation of HC comparisons must consider that oral microbiome composition may be influenced by host‑related factors such as ethnicity, diet, or even geographic location [46]. Moreover, while we acknowledge that the gingiva differs biologically from periapical lesions and that the two tissue types were obtained from different anatomical sites, the HC group still offers useful descriptive information as a general reference for community‑level patterns, although cross‑tissue comparisons should be interpreted with caution, as is appropriate for a cross‑sectional study design. Contamination is an inherent challenge in oral microbiome studies, given that the oral cavity is a heavily colonized environment. All samples were processed using identical pre-extraction disinfection protocols, tissue handling, DNA extraction, and sequencing workflows, which facilitated contamination monitoring and helped minimize technical variability, although low-level contamination from the oral environment, surgical field, or laboratory processing cannot be entirely excluded. This is particularly relevant for interpreting taxa such as *Cutibacterium acnes*, a common skin commensal and recognized contaminant, as the same processing and control procedures were applied to all samples. Furthermore, the gingival data generated herein may serve as a useful reference for future metagenomic investigations of oral soft tissues in health and disease.

The findings of this study have certain potential clinical implications. The microbial taxa and functional pathways associated with persistent apical periodontitis may provide a basis for future diagnostic or prognostic research, although further validation in larger independent cohorts is required before any clinical application can be considered. Future studies integrating multi-omics approaches, such as metabolomics and metatranscriptomics, would help elucidate the host–microbe interactions that underlie lesion chronicity. Additionally, longitudinal follow-up of post-extraction bone healing, particularly using samples obtained at the time of implant placement, would clarify whether residual periapical bacteria are associated with impaired bone healing and inform clinical decision-making regarding extraction site management.

## Conclusion

Shotgun metagenomic analysis showed lower species richness and microbial abundance, but similar predicted functional capacity in persistent apical periodontitis granulomas compared with primary infections. These findings are exploratory and hypothesis‑generating. Future longitudinal cohort studies, together with multi-omics approaches, are needed to further investigate the associations between microbial features and disease persistence.

## Supplementary Material

Supplementary_Material_clean.docx

new Supplementary STROBE checklist cross_sectional.docx new Supplementary STROBE checklist cross_sectional.docx

new_Supplementary_Figure_1.tif new_Supplementary_Figure_1.tif

new_Supplementary_Figure_2.tif new_Supplementary_Figure_2.tif

## Data Availability

The datasets presented in this study can be found in online repositories. The names of the repository and accession number can be found in the article.
