# Supplementary material for: Why does apical periodontitis persist? A shotgun metagenomic survey of the microbial community in primary and persistent periapical granulomas
Source: J Oral Microbiol. 2026 Oct 28;18(1):2741866. doi: 10.1080/20002297.2026.2741866 (PMC13717767; doi:10.1080/20002297.2026.2741866)
Supplement: Supplementary file 1 [file ZJOM_A_2741866_SM7285.docx]

STROBE Statement—Checklist of items that should be included in reports of ***cross-sectional studies***

|  | Item No | Recommendation | Reported in |
| --- | --- | --- | --- |
| **Title and abstract** | 1 | (*a*) Indicate the study’s design with a commonly used term in the title or the abstract | Abstract  Page 2, line 4 |
|  |  | (*b*) Provide in the abstract an informative and balanced summary of what was done and what was found | Abstract  Page 2, lines 8-32 |
| Introduction | | | |
| Background/rationale | 2 | Explain the scientific background and rationale for the investigation being reported | Page 3,  lines 1-37 |
| Objectives | 3 | State specific objectives, including any prespecified hypotheses | Page 3  lines 39-43 |
| Methods | | | |
| Study design | 4 | Present key elements of study design early in the paper | Page 4,  line 4 |
| Setting | 5 | Describe the setting, locations, and relevant dates, including periods of recruitment, exposure, follow-up, and data collection | Page 5,  lines 1-4 |
| Participants | 6 | (*a*) Give the eligibility criteria, and the sources and methods of selection of participants | Page 4, Table 1;  Page 5, lines 1-4 |
| Variables | 7 | Clearly define all outcomes, exposures, predictors, potential confounders, and effect modifiers. Give diagnostic criteria, if applicable | Page 5, Lines 12-20  Page 5, Lines 26-29 |
| Data sources/ measurement | 8* | For each variable of interest, give sources of data and details of methods of assessment (measurement). Describe comparability of assessment methods if there is more than one group | Page 5,  lines 30-36 |
| Bias | 9 | Describe any efforts to address potential sources of bias | Page 5,  lines 26-29 |
| Study size | 10 | Explain how the study size was arrived at | Page 4,  lines 11-25 |
| Quantitative variables | 11 | Explain how quantitative variables were handled in the analyses. If applicable, describe which groupings were chosen and why | Page 7,  lines 25-30 |
| Statistical methods | 12 | (*a*) Describe all statistical methods, including those used to control for confounding | Page 7, Line 13-  Page 8, Line 23 |
|  |  | (*b*) Describe any methods used to examine subgroups and interactions | Not applicable |
|  |  | (*c*) Explain how missing data were addressed | Not applicable |
|  |  | (*d*) If applicable, describe analytical methods taking account of sampling strategy | Page 5, line 39-Page 6, Line8 |
|  |  | (*e*) Describe any sensitivity analyses | Not applicable |
| Results | | | |
| Participants | 13* | (a) Report numbers of individuals at each stage of study—eg numbers potentially eligible, examined for eligibility, confirmed eligible, included in the study, completing follow-up, and analysed | Page8,  lines 27-31 |
|  |  | (b) Give reasons for non-participation at each stage | Not applicable |
|  |  | (c) Consider use of a flow diagram | Not applicable |
| Descriptive data | 14* | (a) Give characteristics of study participants (eg demographic, clinical, social) and information on exposures and potential confounders | Page 8, Table 3 |
|  |  | (b) Indicate number of participants with missing data for each variable of interest | Not applicable |
| Outcome data | 15* | Report numbers of outcome events or summary measures | Not applicable |
| Main results | 16 | (*a*) Give unadjusted estimates and, if applicable, confounder-adjusted estimates and their precision (eg, 95% confidence interval). Make clear which confounders were adjusted for and why they were included | Page 11,  Figure 3  Page 12,  Figure 4 |
|  |  | (*b*) Report category boundaries when continuous variables were categorized | Not applicable |
|  |  | (*c*) If relevant, consider translating estimates of relative risk into absolute risk for a meaningful time period | Not applicable |
| Other analyses | 17 | Report other analyses done—eg analyses of subgroups and interactions, and sensitivity analyses | Not applicable |
| Discussion | | | |
| Key results | 18 | Summarise key results with reference to study objectives | Page 13,  lines 27-32 |
| Limitations | 19 | Discuss limitations of the study, taking into account sources of potential bias or imprecision. Discuss both direction and magnitude of any potential bias | Page 17, Lines 1-  14 |
| Interpretation | 20 | Give a cautious overall interpretation of results considering objectives, limitations, multiplicity of analyses, results from similar studies, and other relevant evidence | Page14, Line 22-,  Page 16, Line 44 |
| Generalisability | 21 | Discuss the generalisability (external validity) of the study results | Page 17,  lines 37-44 |
| Other information | | | |
| Funding | 22 | Give the source of funding and the role of the funders for the present study and, if applicable, for the original study on which the present article is based | Acknowledgments  Page 18,  lines 12-16 |

*Give information separately for exposed and unexposed groups.

**Note:** An Explanation and Elaboration article discusses each checklist item and gives methodological background and published examples of transparent reporting. The STROBE checklist is best used in conjunction with this article (freely available on the Web sites of PLoS Medicine at http://www.plosmedicine.org/, Annals of Internal Medicine at http://www.annals.org/, and Epidemiology at http://www.epidem.com/). Information on the STROBE Initiative is available at www.strobe-statement.org.
