# Supplementary material for: Why does apical periodontitis persist? A shotgun metagenomic survey of the microbial community in primary and persistent periapical granulomas
Source: J Oral Microbiol. 2026 Oct 28;18(1):2741866. doi: 10.1080/20002297.2026.2741866 (PMC13717767; doi:10.1080/20002297.2026.2741866)
Supplement: Supplementary file 2 [file ZJOM_A_2741866_SM7286.docx]

**Supplementary Material**

**Why Does Apical Periodontitis Persist? A Shotgun Metagenomic Survey of the Microbial Community in Primary and Persistent** **Periapical Granulomas**

Yuqing SHEN^a#^, Yiling YANG^a#^, Jian SUN^a^, Zhihui HAN^b^, Yang SUN^a^, Ruixue LI^a^, Yu ZHANG^c^* and Youcheng YU^a^*

^a^ Department of Stomatology, Zhongshan Hospital, Fudan University, Shanghai, China;

^b^ Department of Stomatology, Xuhui Central Hospital, Fudan University, Shanghai, China;

^c^ Department of Preventive Dentistry, Shanghai Ninth People’s Hospital, College of Stomatology, Shanghai Jiao Tong University School of Medicine, Shanghai, China;

*Corresponding authors

1. Youcheng YU, Department of Stomatology, Zhongshan Hospital, Fudan University, 180 Fenglin Road, Shanghai, 200032, China; Email: yu.youcheng@zs-hospital.sh.cn

2. Yu ZHANG, Department of Preventive Dentistry, Shanghai Ninth People’s Hospital, College of Stomatology, Shanghai Jiao Tong University School of Medicine, 639 Zhizaoju Road, Shanghai, 200011, China; Email: zy24ymjulia@163.com

\# These authors contributed equally to this work.

Table S1 Differential abundance analysis results for the top 20 most abundant taxa

| Taxon | *U* | *raw p* | *FDR‑adjusted q* | *r* |
| --- | --- | --- | --- | --- |
| *Porphyromonas gingivalis* | 103 | 0.06399 | 0.467 | 0.31 |
| *Cutibacterium acnes* | 217 | 0.08137 | 0.467 | 0.29 |
| *Actinomyces dentalis* | 169 | 0.822 | 0.9144 | 0.04 |
| *Tannerella forsythia* | 123 | 0.2249 | 0.467 | 0.20 |
| *Prevotella intermedia* | 87 | 0.01737^*^ | 0.467 | 0.39 |
| *Actinomyces israelii* | 165.5 | 0.91 | 1 | 0.02 |
| *Vibrio cholerae* | 166.5 | 0.8845 | 0.9779 | 0.03 |
| *Actinobaculum sp. oral taxon 183* | 132 | 0.3401 | 0.4989 | 0.16 |
| *Staphylococcus aureus* | 137 | 0.3512 | 0.5047 | 0.13 |
| *Actinomyces naeslundii* | 160 | 0.9738 | 1 | 0.01 |
| *Actinomyces sp.* | 122 | 0.1914 | 0.467 | 0.21 |
| *Actinomyces sp. oral taxon 414* | 149 | 0.6877 | 0.7781 | 0.07 |
| *Prevotella nigrescens* | 117 | 0.1362 | 0.467 | 0.24 |
| *Filifactor alocis* | 93.5 | 0.02224^*^ | 0.467 | 0.36 |
| *Porphyromonas gulae* | 108 | 0.06905 | 0.467 | 0.28 |
| *Fusobacterium nucleatum* | 140 | 0.4574 | 0.6076 | 0.11 |
| *Dialister invisus* | 151 | 0.7401 | 0.8303 | 0.06 |
| *Prevotella sp.* | 93 | 0.02275^*^ | 0.467 | 0.36 |
| *[Eubacterium] nodatum* | 96 | 0.02144^*^ | 0.467 | 0.35 |
| *Actinomyces sp. oral taxon 448* | 174 | 0.6411 | 0.7378 | 0.07 |

| KO | Pathway | *U* | *raw p* | *FDR adjusted q* | *r* |
| --- | --- | --- | --- | --- | --- |
| ko01230 | Biosynthesis of amino acids | 85 | 0.01364 | 0.1926 | 0.40 |
| ko00010 | Glycolysis/Gluconeogenesis | 93 | 0.02882 | 0.2131 | 0.36 |
| ko03018 | RNA degradation | 62 | 0.001312 | 0.1282 | 0.53 |
| ko00680 | Methane metabolism | 84 | 0.01131 | 0.1926 | 0.41 |
| ko01501 | beta-Lactam resistance | 98 | 0.03484 | 0.226 | 0.34 |
| ko00710 | Carbon fixation in photosynthetic organisms | 94 | 0.02482 | 0.2131 | 0.36 |
| ko03440 | Homologous recombination | 98 | 0.03484 | 0.226 | 0.34 |
| ko00770 | Pantothenate and CoA biosynthesis | 91 | 0.02309 | 0.2131 | 0.37 |
| ko01212 | Fatty acid metabolism | 105 | 0.04751 | 0.2726 | 0.30 |
| ko04212 | Longevity regulating pathway - worm | 88 | 0.01227 | 0.1926 | 0.39 |

Table S2. Top 10 predicted functional KEGG pathways with nominal differences

Figure S1. Taxonomic composition of bacterial communities across HC, UA, and PA groups at six phylogenetic levels. (a) Phylum level; (b) Class level; (c) Order level; (d) Family level; (e) Genus level; (f) Species level.

**Supplementary Methods**

***Exploratory Random Forest analysis for predictor identification***

To explore potential predictors associated with persistent apical periodontitis, an exploratory random forest analysis was performed incorporating clinical parameters and the relative abundance of key bacterial taxa. To minimize the risk of overfitting given the moderate sample size, 10‑fold stratified cross‑validation (repeated 5 times) and bootstrap resampling (1,000 iterations) was applied. Variable importance was measured using Mean Decrease Accuracy (MDA). Model performance was evaluated using receiver operating characteristic (ROC) curve analysis, and the area under the curve (AUC) was calculated.

**Supplementary Results**

***Exploratory Random Forest Analysis for Biomarkers Exploration***

Exploratory random forest analysis identified swelling duration as the top predictor (MDA = 7.1096). Following clinical factor, the top-ranking bacterial taxa were, in descending order: *Filifactor_alocis, Alloprevotella tannerae, Prevotella buccae, Bacteroides sp. 2_1_22, [Eubacterium] nodatum, Clostridium_sulfidigenes.* These species consistently showed MDA values above 3, highlighting their potential relevance in distinguishing persistent periapical lesions (Figure S2a & S2b). The ROC curve yielded an AUC of 0.69 (95% CI: 0.54-0.84, Figure S2c). Given the exploratory nature of this analysis and the limited sample size, the possibility of model overfitting cannot be excluded. The resulting AUC should be interpreted as exploratory and does not indicate clinical diagnostic performance. These findings should be considered preliminary and hypothesis‑generating rather than definitive. External validation in larger, independent cohorts is needed to determine whether microbial markers can meaningfully improve clinical prediction.

Figure S2 Exploratory random forest analysis of microbial community classification. (a) Proximity matrix scatter plot based on random forest proximity values, reflecting inter-group and intra-group sample similarity. Closer sample points indicate greater similarity in species. (b) Importance ranking of potential predictors identified by random forest. Variable importance was ranked by Mean Decrease Accuracy (MDA), with the top 20 predictors (MDA > 2) considered most informative. (c) Receiver operating characteristic (ROC) curve analysis for evaluating classification model performance. An AUC of 0.69 indicates moderate predictive performance of the model. Given the exploratory nature of this analysis and the limited sample size, these findings should be interpreted with caution.
